# Association analysis between chronic obstructive pulmonary disease and polymorphisms in circadian genes

**DOI:** 10.7717/peerj.9806

**Published:** 2020-08-27

**Authors:** Guo Chen, Jingwei Zhang, Lijuan Zhang, Xuan Xiong, Dongke Yu, Yuan Zhang

**Affiliations:** 1 Department of Geriatrics, Sichuan Academy of Medical Sciences & Sichuan Provincial People’s Hospital, Cheng Du, China; 2 Department of Laboratory Medicine, Chengdu Second People’s Hospital, Cheng Du, China; 3 Personalized Drug Therapy Key Laboratory of Sichuan Province, Sichuan Academy of Medical Sciences & Sichuan Provincial People’s Hospital, Cheng Du, China

**Keywords:** Chronic obstructive pulmonary disease, Circadian genes, Gene polymorphism, Susceptibility factors, Case-control association study

## Abstract

**Background:**

Circadian genes have been suggested to play an important role in lung pathology. However, it remains unknown whether polymorphisms of these genes are associated with chronic obstructive pulmonary disease (COPD). Here, we aimed to investigate the association of circadian genes polymorphisms with COPD in a case-control study of 477 COPD patient and 323 control Han Chinese persons.

**Methods:**

Genotyping assays were carried out for nine single nucleotide polymorphisms (SNPs) from five circadian genes (*PER3*, *CLOCK*, *RORB*, *BMAL1* and *CRY2*) that were previously identified in lung pathology. Age, sex, BMI and smoking status and comorbidities were recorded for all subjects.

**Results:**

No significant association was found in all SNP sites in overall subjects and no significant difference was found in age, sex, smoking status stratification analysis.

**Discussion:**

The findings of this investigation indicated the effect of circadian genes polymorphisms on COPD susceptibility may only be small and possibly dependent on the subject factors, such as age and sex.

## Introduction

Chronobiology is a branch of biology that studies biological rhythms in living organisms. A typical cyclical phenomenon is the ability to respond to infectious disease cycles, and accumulating evidence has proven that various diseases such as cancer, cardiovascular disease, psychiatric disorders and neurodegenerative diseases are associated with chronic mismatch between our life style and biological clock (Badar, 2018; Touitou, Reinberg & Touitou, 2017; Lunn et al., 2017; Smolensky et al., 2016). Chronic obstructive pulmonary disease (COPD) is a progressive lung disease characterized by abnormal inflammation and decline of lung function. It is currently estimated as the fourth leading cause of death worldwide and is projected to be the third leading cause of death by 2020 (https://goldcopd.org/gold-reports/) (Yao et al., 2015). COPD symptoms exhibit circadian variation, with significantly reduced forced vital capacity (FVC), forced expiratory volume in 1 s (FEV1) and peak expired flow at night (Borsboom et al., 1999; Goyal et al., 2020, 2019). In addition, the risk for intubation in the emergency department patients with COPD was found to be elevated during the night or early in the morning (Tsai, Brenner & Camargo, 2007). Evidence also shows that environmental risks of COPD, such as tobacco smoking, can disrupt the biological oscillations, leading to chronic inflammatory responses (Truong et al., 2016). The rhythmic activation and repression of some genes such as *Clock Circadian Regulator* (*CLOCK*), *Aryl Hydrocarbon Receptor Nuclear Translocator Like* (*ARNTL*, also known as *BMAL1*), *Period Circadian Regulator* (*PER*), *Cryptochrome Circadian Regulator* (*CRY*) and *Nuclear Receptor Subfamily 1, group D, member 1* (*NR1D1*, also known as *REV-ERBα*) are recognized as key factors driving physiological circadian oscillations (Sundar et al., 2015a). Sundar et al. (2015b) investigated the circadian molecular clock in lung pathophysiology; their results showed that altered levels of REV-ERBα and RORα (Nuclear Receptor subfamily 1, group F, member 1 (NR1F1)) in mammal’s lung tissue was associated with heightened DNA damage response, cellular senescence and inflammation. Many physiological parameters are regulated by circadian clocks and circadian clock genes, but how these clock molecules influence the development of diseases and impact the genetic predisposition to disease risk are largely unknown. In recent years, positive relationships between single nucleotide polymorphisms (SNPs) in clock genes and diseases, such as cancer, diabetes and mood disorders, has been established, but not yet in COPD. Therefore, we chose to test the SNPs of circadian genes indicated by a search of the literature that might be involved in lung pathology to explore whether they may be linked to risk for COPD in the Chinese Han population.

## Materials and Methods

### Subjects

This study cohort was comprised of 477 patients with mild to moderate COPD (FEV1/FEV1 predicted ≥50%, FEV1/FVC <70%) and 323 controls. The participants were retrospectively recruited from two hospitals: Sichuan Academy of Medical Sciences, Sichuan Provincial People’s Hospital, from 1 June 2018 to 30 November 2019 and Chengdu Second People’s Hospital, from 1 August 2019 to 30 November 2019. COPD was diagnosed based on the subject’s medical history, spirometric data, and post-bronchodilator FEV1/FVC according to the global initiative for chronic obstructive lung disease criteria (GOLD) (De Oca & Pérez-Padilla, 2017). The control group included hospital in-patients admitted due to physical injuries (trauma) and with acute pain caused by lumbar intervertebral disc. For both control and test cases, subjects with “severe” chronic conditions, such as cancer, uncontrollable/stage three hypertension (systolic blood pressure ≥ 180 mmHg or diastolic blood pressure ≥ 110 mmHg), uncontrollable diabetes, chronic kidney failure, cardiovascular disease, osteoporosis, or any mood/psychotic disorders, were excluded from study. The inclusion criteria for controls were: age ≥ 40 years and with no history of chronic pulmonary disease. This study was approved by the Ethics Committee of the Faculty of Medicine, Sichuan Academy of Medical Sciences, Sichuan Provincial People’s Hospital and by the Ethics Committee of Chengdu Second People’s Hospital and was conducted in compliance with the international standards of the journal (Portaluppi, Smolensky & Touitou, 2010). The institutional review board (IRB) approval number was “
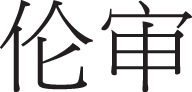
(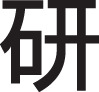) 2018 
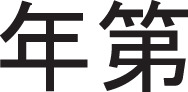
 152 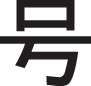
” and “2019076”. Waive of informed consent was granted by the Ethics Committee, as the blood samples used for this study had been collected as routine clinical care of the participants.

### Genotype detection

Selection of the circadian *PER2*, *CLOCK*, *RORB*, *ARNTL* (*BMAL1*), *CRY1* and *CRY2* genes for study was based on putative evidence for lung pathology (Sundar et al., 2015b). Since studies on circadian gene polymorphisms and lung diseases are rare, polymorphisms of these genes were chosen based on reviews assessing circadian system polymorphisms associated with other pathologies, including cancer, metabolic diseases and mood disorders (Valenzuela et al., 2016). The SNPedia and PubMed databases were used to search for SNPs with positive associations with diseases. The dbSNP and 1000 Genome databases were used to identify the minor allele frequency (MAF) in East Asian, and only MAF > 0.05 SNPs were selected. Primary information of these SNPs is shown in Table 1.

**Table 1:** Characteristics of circadian genes and SNPs in this study.

SNP	Chr	Position	Ref	Alt	Gene	Region	Related phenotype	1000 Genome (%)
rs934945	2	239155053	C	T	*PER2*	Exonic	Soft tissue sarcoma (Benna et al., 2018), Cardiovascular disease (Skrlec et al., 2019), Metabolic Syndrome (Englund et al., 2009), Psychotic Disorder (Liu et al., 2015)	AFR: 1
								EAS: 26
								EUR: 18
rs1048004	4	56300209	C	A	*CLOCK*	UTR3	Breast cancer (Hoffman et al., 2010b)	AFR: 15
								EAS: 10
								EUR: 31
rs3805151	4	56337041	T	C	*CLOCK*	Intronic	Breast cancer (Hoffman et al., 2010b), Mood disorder (Sjoholm et al., 2010)	AFR: 17
								EAS: 57
								EUR: 34
rs3750420	9	77249382	C	T	*RORB*	Intronic	Breast cancer (Zienolddiny et al., 2013), Mood disorder (Partonen, 2012)	AFR: 16
								EAS: 52
								EUR: 30
rs3903529	9	77264228	T	A	*RORB*	Intronic	Breast cancer (Zienolddiny et al., 2013), Mood disorder (Partonen, 2012)	AFR: 11
								EAS: 9
								EUR: 24
rs969485	11	13403043	G	A	*BMAL1*	Intronic	Breast cancer (Zienolddiny et al., 2013), Type 2 diabetes (Woon et al., 2007)	AFR: 51
								EAS: 43
								EUR: 72
rs2290035	11	13407771	T	A	*BMAL1*	Intronic	Breast cancer (Zienolddiny et al., 2013), Type 2 diabetes (Woon et al., 2007)	AFR: 30
								EAS:26
								EUR: 57
rs11038689	11	45874264	A	G	*CRY2*	Intronic	Breast cancer and non-Hodgkin’s lymphoma (Hoffman et al., 2010a; Hoffman et al., 2009)	AFR: 12
								EAS: 12
								EUR: 22
rs1056560	12	107385610	C	A	*CRY1*	UTR3	Gastric cancer (Qu et al., 2016)	AFR: 32
								EAS: 70
								EUR: 57

**Notes:**

SNP, single nucleotide polymorphism.

Chr, chromosome.

Ref, reference allele.

Alt, alternative allele.

1000 Genome: the alternative allele frequency in three different populations: AFR (African), EAS (East Asian), EUR (European) from 1000 Genome (https://www.internationalgenome.org/).

Genomic DNA was extracted from whole venous blood samples that had been earlier drawn from participants using a whole blood genomic DNA extraction and purification kit (Cat. No.: DP348-03; Tiangen, China). All genotyping experiments were done by the Genesky Bio-pharm Technology Company, using SNPscan technology (Hu et al., 2018; Ting et al., 2006). For quality control, ~5% of samples were genotyped in duplicate, and the results indicated 100% consistent.

### Statistical analysis

Data regarding age are presented as mean ± SD, with between-group comparison done by Student’s *t*-test with two-tailed values. The variables of sex and smoking status are presented as percentages and are compared using the Chi-squared test. The Hardy–Weinberg equilibrium test was performed on the data of the control groups using the exact test to evaluate the reliability of the control group. As there were significant differences in the age and sex between cases and controls, an unconditional logistic regression model adjusting for age, sex, BMI, smoking status and comorbidities were used to evaluate the effect of the SNP outcome by Odds ratio (OR) and 95% Confidence interval (CI). To comprehensively analyze the association between the individual SNPs and COPD risk, we adopted four genetic models: the additive effect model, the dominant effect model, the recessive effect model and the allelic effect model. PLINK (1.0.7) and *R* (3.5.1) were used for statistical analyses. A *P* < 0.05 was considered to indicate statistical significance. Power calculation (α = 0.05) was performed using Power and Sample Size Calculation Software (3.6.1).

## Results

### Characteristic information of the study population

The basic information of the patients and controls is summarized in Table 2. A total of 800 subjects were eligible for analysis. There were 477 COPD cases and 323 controls, 501 males and 299 females. There were significant differences between cases and controls with regards to sex, age and smoking status (Table 2), therefore, unconditional logistic regression test was applied to analyze the association between the SNPs and risk of COPD.

**Table 2:** Clinical features for COPD patients and controls in this study.

	Case (*n* = 477)	Control (*n* = 323)	*P* value
Male	343	158	7.01 × 10^−11^
Female	134	165	
Age (Mean ± SD)	75.92 ± 10.21	72.64 ± 10.40	1.29 × 10^−5^
Age ≤ 60 (Mean ± SD)	55.53 ± 4.05 (*n* = 20)	55.15 ± 4.62 (*n* = 34)	0.77
61~70 (Mean ± SD)	66.03 ± 2.68 (*n* = 127)	65.95 ± 2.85 (*n* = 107)	0.82
71~80 (Mean ± SD)	75.88 ± 2.98 (*n* = 153)	75.89 ± 2.88 (*n* = 104)	0.99
81~90 (Mean ± SD)	84.89 ± 2.70 (*n* = 155)	88.88 ± 2.75 (*n* = 70)	0.98
≥91 (Mean ± SD)	93.13 ± 3.06 (*n* = 22)	92.91 ± 2.77 (*n* = 8)	0.87
BMI	22.74 ± 4.03	23.59 ± 4.37	0.30
Hypertension (%)	60	45	0.70
Type 2 Diabetes (%)	33	21	0.94
Hyperlipidemia (%)	26	13	0.48
Smoker + ex-smoker	271	45	2.20 × 10^−16^
Non-smoker	175	266	

### Genotype and allele distribution of circadian gene polymorphisms and associations with COPD

We screened five circadian genes and nine SNPs in 477 COPD patients and 323 controls. One of the SNPs, rs3903529 showed deviation from Hardy–Weinberg equilibrium in the control group (*P* < 0.05); therefore, this SNP was excluded from further analyses. The association between these SNPs and COPD risk was assessed in logistic regression analysis adjusted by age, sex, smoking status and comorbidities in four different genetic models (Allelic, Additive, Dominant and Recessive). None of these SNPs reached statistical significance (Table 3). Last, we analyzed these SNPs with respect to age (≤60, 61~70, 71~80, 81~90, ≥91), sex (male, female) and smoking status (smokers + ex-smokers and non-smokers) stratification by logistic regression in four different genetic models. No reasonable conclusion was drawn in the age group younger than 60 years and older than 90 years due to very limited sample size. No significant relationship was found in the other age groups and sex stratification analysis although there was a weak association observed in rs1103868 in the 71~80 age group (OR = 1.86, 95% CI [1.04–3.34] (Additive, *P* = 0.04) and OR = 2.00, 95% CI [1.08–3.71] (Dominant, *P* = 0.03 )), and in rs3750420 in the male group (OR = 1.36 and 95% CI [1.03–1.79] (Additive, *P* = 0.03) and OR = 1.96 and 95% CI [1.12–3.42] (Recessive model, *P* = 0.02)). However, the sample size of age group 71~80 (153 vs. 104) was underpowered for rs1103868 and both of the associations became insignificant after multiple test correction (Table 4). None of these SNPs were associated with COPD when smoking status was considered. To determine whether the sample size of this study has power to detect the real difference, a power analysis was performed. From the result of Table 5, the sample size of 477 vs. 323 ensured that a two-sided test with α = 0.05 had 80% power to detect an relative risk of 2.0 (Ψ = 2.0).

**Table 3:** Association analysis between circadian related gene polymorphisms and COPD risk in an allelic/additive/dominant/recessive genetic model.

SNP	Gene	Type of test^#^	Number of genotypes in cases (dd/Dd/DD)	Number of genotypes in controls (dd/Dd/DD)	OR^^^	L95	U95	*P*	Adjusted P*
rs934945	*PER2*	Allelic	36/188/253	29/138/156	0.86	0.69	1.07	0.19	>0.99
		Additive			0.86	0.67	1.12	0.27	>0.99
		Dominant			0.84	0.60	1.16	0.28	>0.99
		Recessive			0.83	0.45	1.50	0.53	>0.99
rs1048004	*CLOCK*	Allelic	3/63/411	2/44/277	0.97	0.66	1.42	0.92	>0.99
		Additive			0.76	0.49	1.19	0.23	>0.99
		Dominant			0.77	0.48	1.23	0.27	>0.99
		Recessive			0.46	0.06	3.4	0.45	>0.99
rs3805151	*CLOCK*	Allelic	73/226/178	55/152/116	0.94	0.77	1.15	0.53	>0.99
		Additive			0.87	0.69	1.11	0.87	>0.99
		Dominant			0.81	0.58	1.14	0.23	>0.99
		Recessive			0.88	0.56	1.37	0.57	>0.99
rs3750420	*RORB*	Allelic	93/221/163	53/155/115	1.09	0.90	1.34	0.38	>0.99
		Additive			1.11	0.88	1.40	0.38	>0.99
		Dominant			1.04	0.73	1.46	0.84	>0.99
		Recessive			1.35	0.88	2.07	0.17	>0.99
rs969485	*BMAL1*	Allelic	80/234/163	66/159/98	0.86	0.70	1.05	0.14	>0.99
		Additive			0.91	0.72	1.14	0.40	>0.99
		Dominant			0,91	0.64	1.30	0.61	>0.99
		Recessive			0.83	0.54	1.26	0.38	>0.99
rs2290035	*BMAL1*	Allelic	29/193/255	22/129/172	0.98	0.78	1.23	0.83	>0.99
		Additive			0.96	0.73	1.25	0.74	>0.99
		Dominant			1.03	0.75	1.44	0.83	>0.99
		Recessive			0.64	0.33	1.26	0.20	>0.99
rs11038689	*CRY2*	Allelic	6/118/353	5/74/244	1.06	0.78	1.43	0.71	>0.99
		Additive			1.04	0.73	1.47	0.84	>0.99
		Dominant			1.11	0.76	1.62	0.61	>0.99
		Recessive			0.41	0.09	1.75	0.23	>0.99
rs1056560	*CRY1*	Allelic	38/206/233	28/147/148	0.91	0.73	1.14	0.42	>0.99
		Additive			0.99	0.76	1.27	0.94	>0.99
		Dominant			0.71	0.51	0.99	0.05	0.45
		Recessive			0.77	0.42	1.42	0.41	>0.99

**Notes:**

# “D” represent wild type and “d” represent mutant type. Allelic: d vs. D; Additive: dd vs. Dd vs. DD; Dominant: dd + Dd vs. DD; Recessive: dd vs. Dd + DD.

^ Logistic regression results were adjusted for age, sex, BMI, smoking status (smoker + ex-smoker and non-smoker), comorbidities (hypertension, type 2 diabetes and hyperlipidemia).

* Corrected by Bonferroni’s method.

SNP, single nucleotide polymorphism.

OR, odds ratio.

L95, lower bound of 95% confidence interval for odds ratio.

U95, upper bound of 95% confidence interval for odds ratio.

**Table 4:** Significant SNPs in stratification analysis.

Stratification Class	SNP	Gene	Minor Allel	Test^#^	Total number of samples	OR^^^	L95	U95	*P*	Adjusted P*
Males	rs3750420	*RORB*	C	Allelic	501	1.36	1.03	1.79	0.03	0.24
		*RORB*	C	Additive	501	1.36	1.03	1.79	0.03	0.24
		*RORB*	C	Dominant	501	1.30	0.88	1.92	0.20	>0.99
		*RORB*	C	Recessive	501	1.96	1.12	3.42	0.02	0.16

**Notes:**

# “D” represent wild type and “d” represent mutant type. Allelic: d vs. D; Additive: dd vs. Dd vs. DD; Dominant: dd + Dd vs. DD; Recessive: dd vs. Dd + DD.

^ Logistic regression results in the Male group, the results were adjusted for age, BMI, smoking status (smoker + ex-smoker and non-smoker), comorbidities (hypertension, type 2 diabetes and hyperlipidemia).

* Corrected by Bonferroni’s method.

SNP, single nucleotide polymorphism.

OR: odds ratio.

L95, lower bound of 95% confidence interval for odds ratio.

U95, upper bound of 95% confidence interval for odds ratio.

**Table 5:** Power of the study at current sample size.

SNP	Type of test	Alt^#^	Ψ*	Power	Required sample size (cases vs. controls)	Whether the present study met the sample size requirement (YES/NO)
rs934945	Allelic	0.26	2.0	0.8	186 vs. 126	YES
rs1048004	Allelic	0.10	2.0	0.8	355 vs. 241	YES
rs3805151	Allelic	0.57	2.0	0.8	179 vs. 122	YES
rs3750420	Allelic	0.52	2.0	0.8	170 vs. 116	YES
rs969485	Allelic	0.43	2.0	0.8	163 vs. 111	YES
rs2290035	Allelic	0.26	2.0	0.8	186 vs. 126	YES
rs11038689	Allelic	0.12	2.0	0.8	307 vs. 209	YES
rs1056560	Allelic	0.70	2.0	0.8	226 vs. 154	YES

**Notes:**

# The alternative allele frequency in East Asian (EAS) from 1000 Genome (https://www.internationalgenome.org/).

* The odds ratio of exposure in cases relative to controls.

### Haplotype analysis

Pairwise linkage disequilibrium (LD) was assessed for the SNPs within the same chromosome. Overall, LD was low among the SNPs, with the highest *R*^2^ being 0.47 between rs969485 and rs2290035 (Table 6). Due to most of the SNPs situated in the different chromosomes, and the LD was low, performing haplotype analysis was not considered necessary (Hodoglugil, Williamson & Mahley, 2010).

**Table 6:** Linkage disequilibrium results for the SNPs within the same gene.

Chr	BP	SNP	*R*^2^*
11	13381496	rs969485	0.47
	13386224	rs2290035	

**Notes:**

* As default set by Plink, only *R*^2^ > 0.2 was reported.

Chr, chromosome.

BP, physical position (base-pair).

SNP, single nucleotide polymorphism.

## Discussion

In this case-control study, we investigated the potential association between nine SNPs from five different circadian genes and the COPD risk in the Chinese Han population. The overall result didn’t show any significant association between these SNPs and COPD risk or in the age, sex and smoking status stratification analysis.

The circadian oscillator is located in the anterior hypothalamus and controls circadian processes, though peripheral tissue, including the lung also shows cell autonomous oscillators, responsive to environmental stimuli (Sundar et al., 2015b). The core clock genes that drive circadian-related feedback loops include CLOCK, CRY1, CRY2, PER1, PER2 and PER3; of these CLOCK acts as a positive regulator, while the remaining five genes act as negative regulators (Lee et al., 2015). The heterodimer of the transcription factors CLOCK and BMAL1 regulates the transcription of *PER* and *CRY* genes via E-box sequences within their promoters. PER and CRY heterodimerize and block E-box-dependent transcriptional activity of the CLOCK: BMAL1 complex. The counteracting activity of REV-ERBs and ROR mediate BMAL1 expression and CHRONO, a transcriptional repressor that acts independently of the circadian transcriptional repressor such as CRY1 and CRY2, can directly interact with BMAL1 and negatively regulate circadian oscillations (Takahashi, 2015). An environmental exposome, such as cigaret smoking, could influence the lung cells and macrophages through activations of kinases that lead to posttranslational modification of molecular clock proteins (Sundar et al., 2015b). Theoretically, dysfunction of the clock-controlled genes in the bronchial epithelial cells can cause internal desynchrony, contributing largely to clock-dependent pathophysiology of chronic airway diseases (Sundar et al., 2015b). Animal studies have shown that after cigaret smoking or lipopolysaccharide inducement, cytokine or neutrophil levels were significantly higher in *BMAL1* knockout mice compared with controls (Gibbs et al., 2014; Jaradat et al., 2006). In addition to alteration in the environmental stimuli-induced defense, theoretically circadian rhythm dysfunction could also influence the pulmonary function by changing timing signals through sympathetic and parasympathetic innervation (Buijs & Kalsbeek, 2001). Notably, genetic variants in clock genes have been reported to be associated with susceptibility to cancer, metabolic diseases, and psychotic and mood disorders (Gu et al., 2015). However, there are no published association studies evaluating the circadian gene polymorphism and chronic pulmonary disease, and this study is the first study to investigate any potential association between clock gene polymorphisms and COPD risk.

In this case-control study, overall analyses showed that no significant SNPs were found to be associated with COPD. However, in the stratification analysis, a weak positive association was observed in *RORB* rs3750420 in the male subjects, but this association became insignificant after adjusting for multiple comparisons. In our opinion, larger samples are needed to properly explore this hypothesis. Abundant researches have shown that the chronotype, which is the diurnal preferences that manifest in personal sleep-wake rhythms, differs in females and males as well as in young and elderly, and this difference, at some point, depends on the variants of “clock” genes (Roenneberg et al., 2007). The expression of circadian genes *PER2*, *PER3* and *ARNTL1* show differences with sex in human cerebral cortex and much earlier expression timing of these three genes in women than in men (Lim et al., 2013). Recent studies on psychiatric disorder also found a relationship between *PER3 VNTR* genotype and age at onset of bipolar disorder (Benedetti et al., 2008). The minor allele of *CLOCK* gene polymorphism 3111T/C was associated with higher susceptibility of Alzheimer’s disease only in APOE ε4 carriers (Yang et al., 2013). However, low significance may also be due to the relatively smaller numbers of subjects and variations in the patient population. Hence, a larger sample size of both groups should be considered in the future to further validate the results.

There are several limitations in our current study. Firstly, the SNPs we chose from genes involved in lung pathology were based on literature reporting positive relationship with other disorders rather than respiratory disease, per se, due to scarce evidence of association of circadian gene polymorphism with lung disease. Therefore, it is possible that we missed finding out potentially important lung-disease-related SNPs because of literature renewal lag or genotyping method limitations. Secondly, this study did not explore enough circadian rhythmic phenotypes, such as sleep patterns and pulmonary functions, which could also be linked to these SNPS. Circadian rest-activity rhythm disturbance, such as insomnia, dyspnea and nocturia, is commonly seen in COPD patients. Disparity from normal of the 24 h rest-activity rhythm of COPD subjects has been reported for the activity parameters of mean intradaily variability, interdaily stability, mean of 10 consecutive hours of highest activity, and mean of five consecutive hours of lowest activity. These measures were confirmed by previous studies to be significantly different in COPD patients compared with controls (Nunes et al., 2017), but whether they are associated with circadian gene polymorphisms remains unclear. As these tests were not available during this investigation, further prospective studies are needed to investigate the linkage of these measures to circadian gene polymorphisms and whether they are associated with COPD.

## Conclusion

In conclusion, no overall relationship between circadian gene polymorphisms and COPD was detected in the Chinese Han population. A larger cohort investigation is required to gain evidence within each stratum to validate that whether the circadian gene polymorphisms are associated with COPD only in a sex or age stratification. Additionally, as the alternative allele frequency distribution varies among different ethnic groups, which would influence the susceptibility of the disease (Lee et al., 2003), a validation of these SNPs across the ethnicities may be need in the future.

## Supplemental Information

10.7717/peerj.9806/supp-1 Supplemental Information 1 Raw data: the subjects’ genotypes, age, sex, BMI, smoking status and comorbidities for statistical analysis.

## Additional Information and Declarations

### Data Availability

The following information was supplied regarding data availability: The raw data is available as a Supplemental File.
